# Utility of Preoperative CA125 Assay in the Management Planning of Women Diagnosed with Uterine Cancer

**DOI:** 10.1155/2014/497478

**Published:** 2014-02-27

**Authors:** N. Povolotskaya, N. Das, K. Dhar, D. Brinkmann, F. Gardner, R. Woolas

**Affiliations:** Portsmouth Cancer Centre, Queen Alexandra Hospital, Southwick Hill Road, Cosham, Portsmouth, Hampshire PO6 3LY, UK

## Abstract

*Objective.* This study assesses the role of preoperative serum CA125 levels in the planning treatment options for women diagnosed with uterine cancer. *Material and Method.* Ninety five consecutive patients diagnosed with uterine cancer during a four-year period were identified. Age ranged from 35 to 89 years with a mean age of 69 years. The preoperative CA125 levels were dichotomised at 28 U/mL (using ROC analysis to identify the best discriminating threshold for 5-year survival). This level was then correlated with preoperative prognostic indicators: patient age, tumour grade, and histopathological tumour cell type. Survival data was plotted using Kaplan-Meier curves and analysed using the log-rank test. Univariate and multivariate analysis were performed to identify the predictors of overall survival. *Results.* The mean age of patients was 69 years (range: 35–89). On univariate analysis, the use of preoperative CA125 levels of greater or less than 28 U/mL correlated significantly with age (*P* = 0.01), the grade of disease (*P* = 0.02) and unfavourable tissue type (*P* = 0.03). This threshold CA125 level had a sensitivity of 75%, specificity of 76%, positive predictive value of 35% and negative predicative value of 96.25%, and a likelihood ratio of 3.12 for predicting nodal disease. Using a threshold of preoperative CA125 level of 28 U/mL (area under curve: 0.60) was also a significant predictor of 5-year survival (log-rank test, *P* = 0.01). Using Cox multivariate survival analysis to identify predictive preoperative factors overall, unfavourable cell type was the strongest predictor of survival (Chi square = 36.5, df = 4, and *P* = 0.001), followed by preoperative CA125 level (CA125 > 28 U/mL, *P* = 0.011) and unfavourable preoperative grade (*P* = 0.017). Amongst patients with a favourable histological tissue type (endometrioid), preoperative CA125 levels predicted overall survival (Chi square = 6.039, df = 2, *P* = 0.02); however unfavourable preoperative grade did not (*P* = 0.5). Overall, at five-year follow-up, while there were no deaths among the women with preoperative serum CA125 less than 12 U/mL, eleven of the twenty-three deaths (47.82%) in the study occurred in women with a preoperative CA125 more than 28 U/mL. *Conclusions.* A preoperative CA125 assay for women with uterine cancer is a relatively inexpensive, reproducible, and objective test which provides valuable information regarding the risk of metastatic disease and overall likelihood of long term survival. Patients with a low likelihood of metastatic/nodal disease (favourable tissue type and CA125 level < 28 U/mL) and significant comorbidities may benefit from avoiding an extended complete staging procedure. Alternatively, a high level of CA125 may prompt further imaging and multidisciplinary discussions to plan for individualised management and consideration for recruitment to clinical trials.

## 1. Introduction

Endometrial cancer is the fourth most common cancer in women in the UK with 7,703 cases diagnosed in 2008 accounting for 5% of all female cancers [1–3]. Estimated life time risk of developing uterine cancer for women in the UK is 1 in 46 [4]. The number of cases diagnosed annually is expected to rise [5, 6]. The rise in deaths follows the increase in the number of women being diagnosed with uterine cancer, with its incidence rising by 43 percent since the mid-1990s, from 13.7 to 19.6 per 100,000 [3]. Although survival from uterine cancer continues to improve gradually, more women are in fact dying from the disease, because of the rise in numbers of women being diagnosed [7].

Many women presenting with endometrial cancer are elderly [3, 8] and often have significant co-morbidities. Patients who present with disease confined to the uterus have a good prognosis while those with advanced stage disease have a higher risk of recurrence and death. Since 1988 the International Federation of Gynaecology and Obstetrics (FIGO) has recommended a complete surgical staging (last revised in 2009 [9]) as the initial treatment for women diagnosed with corpus cancer [10]. A complete surgical staging is not an option for a group of women with significant co-morbidities that increase their risk for adverse intraoperative and post-operative outcomes. Individualisation of the extent of surgical staging procedure for women with uterine cancer requires a careful appraisal of preoperative prognostic information and a flexible approach on the part of the appropriately trained surgeon [11].

At present patient age, histological cancer type [12–14], preoperative grade [15, 16], imaging evidence of degree of myometrial invasion [17–20], and the presence of metastatic disease are used to predict the group of patients with high risk of recurrence preoperatively and optimise individualised treatment accordingly [11, 21, 22]. Unfortunately none of these features are able to predict the metastatic potential of the disease with certainty. Preoperative grade of disease following an outpatient biopsy by pipelle sample or endometrial curettage is inaccurate, with up to forty percent of the patients being upgraded after surgery [23–29]. Preoperative imaging techniques, used to identify myometrial invasion and metastatic disease, have similar problems; they are often expensive, have varied accuracy, and may not be available universally [18, 30]. New molecular markers techniques (e.g., loss of ER/PR expression [31], P16 expression, high expression of P53 [32, 33], high proliferative rate (Ki-67) [34], high expression of stathmin [35], overexpression of Her2NEU [36], and aneuploidy [37]) have been studied in hysterectomy and preoperative uterine biopsy with a high degree of correlation between the results. However practical clinical use of these markers awaits further evaluation.

Since the discovery of CA125 in 1981 by Bast et al. [38, 39] there have been a number of reports about this glycoprotein and its role in endometrial cancer [40]. Niloff initially reported elevated levels of CA125 in patients with advanced stage and recurrent endometrial cancer [41]. Several reports then followed reporting elevated levels in both primary and recurrent endometrial cancer [42–56]. In a retrospective review of 210 women with endometrial cancer, Sood et al. found elevated serum CA125 levels to be a strong predictor of extrauterine disease and mortality. A management algorithm based on preoperative CA125 levels and the validity of the use of the vaginal hysterectomy in a specific group of patients with endometrial cancer was also presented [57–59]. Clinically it will be extremely useful to have an inexpensive and objective test for preoperative identification of the patients, who have a high likelihood of extrauterine disease and poor prognosis for overall survival. Such a test could be utilised to determine what preoperative investigations are appropriate and what treatment options may be suitable.

This study evaluated preoperative CA125 levels as a marker of metastatic potential and predictor of overall long term survival in patient with uterine cancer. This study also explores the role of CA125 in identifying women who may benefit from various treatment options available for women with this disease.

## 2. Materials and Method

During a four-year period (1997–2000) patients with the diagnosis of uterine cancer were identified from a prospectively collected data base at the St. Mary's Hospital, Portsmouth. All our patients were staged according to the FIGO guidelines. Following the diagnosis, patients underwent surgical staging which included a total abdominal hysterectomy, bilateral salpingo-oophorectomy, omental biopsy, and peritoneal washings with or without lymphadenectomy depending on preoperative risk factors. Adjuvant treatment where indicated was recommended following multidisciplinary discussions and as per departmental protocols. All patients had their serum CA125 levels measured as part of preoperative assessment. Demographic, operative, histopathological data were documented prospectively and five-year survival data (disease related morbidity) was collected from the oncology data base or review of the case notes as required. Receiver Operative Curve (ROC) analysis was used to identify the best discriminating threshold of the preoperative serum CA125 levels for overall survival. This level was then correlated with the tumour grade, cell type, lymph node involvement, and the stage of the disease. For the purpose of analysis histopathological tissue type was categorized into favourable (endometrioid) and unfavourable (squamous, clear cell, carcinosarcoma, and serous) groups. Survival data was plotted using Kaplan-Meier curves and analysed using a log-rank test. Univariate and multivariate analysis (Cox proportional hazard) were performed to identify predictors of overall survival as well. All analyses were performed using SPSS version 11 statistical package.

## 3. Results

**Figure 1:**
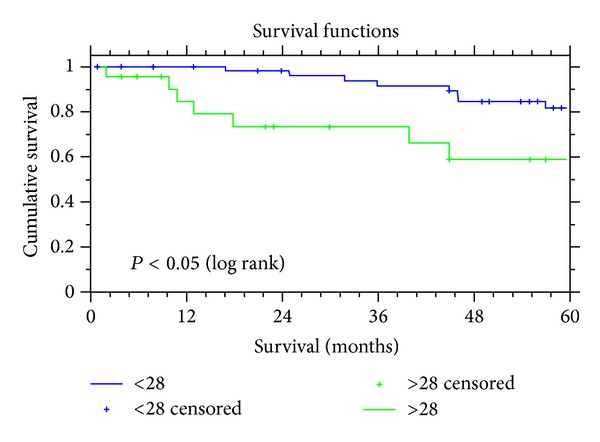
Kaplan Meier survival curves and dichotomized CA125 levels.

Ninety-five patients diagnosed with uterine cancer were identified during a four-year period of time (1997–2000). Ages ranged from 35 to 89 years with a mean 69 years. All patients underwent surgical staging which included a total abdominal hysterectomy, bilateral salpingo-oophorectomy, omental biopsy, and peritoneal washings; 32% of the patients with either unfavourable tissue type or high tumour grade on preoperative histology underwent a pelvic +/− para-aortic lymphadenectomy as well.  Table 1 shows the distribution of patients with respect to age, preoperative grade, and stage of the disease. CA125 levels ranged from 4 to 975 U/mL (with a median of 19 U/mL). Patients were divided into two groups based on preoperative CA125 levels (</> 28 U/mL) following a ROC analysis. On univariate analysis using a threshold of preoperative CA125 levels (at 28 U/mL) correlated with older age group (>80 years), unfavourable tumour grade (poorly differentiated), unfavourable tissue type (serous, clear cell adenosquamous, and carcinosarcoma), nodal status, and overall survival at 5 years (Table 2). A preoperative CA125 level of more than 28 U/mL had a sensitivity of 75%, specificity of 76%, a positive predictive value of 35% and negative predictive value of 96.25%, and a likelihood ratio of 3.12 for predicting nodal disease. Use of threshold of preoperative CA125 level of 28 U/mL was also a significant predictor of five year survival (log-rank test, *P* = 0.01) (Figure 1) along with higher risk age group (>80 years, *P* = 0.01) and unfavourable preoperative grade (log-rank test, *P* < 0.05).

Following Cox multivariate survival analysis to identify preoperative predictive factors overall, unfavourable tissue type was the strongest predictor of overall long term survival (Chi square = 36.5, df = 4, and *P* = 0.001) followed by preoperative CA125 (CA125 > 28 U/mL, *P* = 0.011) and unfavourable preoperative tumour grade (*P* = 0.017). Interestingly, among the patients with favourable tissue type (endometrioid), preoperative threshold of CA125 level continued to predict overall survival (Chi square = 6.039, df = 2, and *P* = 0.02) but amongst unfavourable preoperative tumour grade CA125 was a poor predictor of overall survival (*P* = 0.5).

There were no deaths among women with a preoperative serum CA125 less than 12 U/mL; however eleven of the twenty-three deaths (47.8%) at five-year follow-up occurred in women with a preoperative CA125 of more than 28 U/mL.

## 4. Discussion

Revised in 2009 FIGO recommendations on initial surgical management of uterine cancer involve a total hysterectomy and bilateral salpingo-oophorectomy. The decision to perform an extended staging with pelvic and para-aortic lymphadenectomy is based on available preoperative data on high risk prognostic variables such as patient age, high grade tumour, and unfavourable histological cancer type. It is widely accepted that high grade disease is associated with up to 30% of retroperitoneal nodal involvement [60]. The rational for the extended surgery is to assign a more accurate stage to the disease, plan adjuvant therapy accordingly, and offer the patients recruitment into appropriate clinical trials. Additionally, there is a body of evidence which suggests that lymphadenectomy in this group of patients is not only of diagnostic but also of therapeutic benefit [61–67]. Unfavourable histopathological tissue types of uterine cancer are associated with poor prognosis, but they are infrequent generally accounting for less than 10% of the patients [68, 69].

The majority of endometrial cancers are diagnosed following an office endometrial biopsy or a diagnostic hysteroscopy; however preoperative grade is often upgraded postoperatively in up to 40% of cases in some reported series [23–25, 27–29].

Another tool for preoperative identification of the metastatic potential of the disease would be useful to help to identify the patients who would benefit from a full retroperitoneal assessment of nodal disease status. It may also help to avoid extensive staging procedures in women with multiple comorbidities when the disease has low potential for recurrence without adversely affecting survival.

There is evidence to suggest that CA125 levels are useful in the management and follow-up of women with uterine cancer. Elevated CA125 levels are not only associated with metastatic disease but recurrent disease as well [50, 70–72].

On review of the available data it appears that the threshold cut-off level for a normal serum CA125 in women with uterine cancer is lower than the traditionally accepted threshold level of 35 U/mL as used for other clinical settings like ovarian cancer [50, 56, 71–73].

In 1994, Alagoz et al. first suggested that a lower threshold of CA125 would be appropriate for use in endometrial cancer [74]. Recent studies have suggested that a cut-off level of 20 U/mL may be appropriate threshold to identify extrauterine spread [50]. Some other studies used the cut-off of 35 U/mL [43, 54, 75]. Unfortunately there is no study with a large number of participants and no widely accepted threshold level of CA125 to base practice on.

The analysis in this study suggests adopting a threshold level of 28 U/mL. This is consistent and within the range of the CA125 threshold levels as suggested by the other investigators.

A preoperative CA125 assay among the women with uterine cancer correlated with known prognostic variables such as age, cell type, and stage and overall long term survival. At a CA125 level of more than 28 U/mL, there was a three times greater likelihood of identifying nodal disease and the negative predictive value for nodal disease at a level below 28 U/mL was 95% among our patients. This is as good as the reported rates of other investigators using more expensive and variably available sophisticated imaging modalities. With regard to a long term survival, threshold of preoperative CA125 level of 28 U/mL correlated with overall five-year survival and continued to do so in the favourable (endometrioid) cell type as well.

A preoperative CA125 assay for women with uterine cancer is a simple, inexpensive, reproducible, and objective test which provides additional information regarding the risk of metastatic disease and ultimate prognosis (long term survival). It provides useful information to clinicians, which could help to individualize and tailor treatment accordingly. Patients with a low likelihood of metastatic/extrauterine disease (favourable histopathological tissue type and CA125 < 28 U/mL in the presented data) and significant comorbidities may also benefit from consideration of vaginal hysterectomy or alternative treatment options rather than an extensive surgical staging procedure. Alternatively, a CA125 above 28 U/mL may prompt further imaging (to clarify presence or absence of extrapelvic disease) and multidisciplinary discussions as to the best of management plan for the individual (radical surgery/neoadjuvant/adjuvant therapy). It will also facilitate consideration of patients for recruitment to clinical trials.

We therefore feel that a preoperative CA125 level is a valuable preoperative tool which may be utilised to individualise the treatment offered for uterine cancer. Additionally, in the age of cancer waiting time targets and limited available resources, such as in the National Health Service in the United Kingdom, a preoperative CA125 assay will help the clinicians to allocate clinical resources accordingly.

## Figures and Tables

**Table 1:** Characteristics of patients with endometrial cancer *N* = 95.

Characteristics	*N* (%)
Age > 80 years	19 (20.2)
Unfavourable histopathological tissue type	15 (15.8)
Unfavourable preoperative grade	14 (14.7)
CA125 > 28 U/mL	28 (29.5)

**Table 2:** Correlation with elevated CA125 (>28 U/mL).

Variable	Odds ratio	CI	*P* value
Age > 80 years	1.1	0.9–1.5	0.01
Unfavourable histopathological tissue type	1.5	1.1–2.1	0.03
Unfavourable preoperative tumour grade	1.2	0.9–1.5	0.02
Nodal involvement	1.4	0.9–2.3	0.03
5-year survival	2.2	0.8–5.6	0.01

(CI: confidence interval; *P* value based on Pearson Chi square).
